# *Pseudomonas* cold shock proteins suppress bacterial effector translocation in *Nicotiana benthamiana*

**DOI:** 10.3389/fmicb.2025.1539906

**Published:** 2025-01-23

**Authors:** Shen Cong, Jun-Zhou Li, Mei-Ran Zhang, Hai-Lei Wei, Wei Zhang

**Affiliations:** ^1^State Key Laboratory of Efficient Utilization of Arid and Semi-arid Arable Land in Northern China, Key Laboratory of Microbial Resources Collection and Preservation, Ministry of Agriculture and Rural Affairs, Institute of Agricultural Resources and Regional Planning, Chinese Academy of Agricultural Sciences, Beijing, China; ^2^School of Chemistry and Biological Engineering, University of Science and Technology Beijing, Beijing, China; ^3^Plant Pathology and Plant-Microbe Biology Section, School of Integrative Plant Science, Cornell University, Ithaca, NY, United States

**Keywords:** *Pseudomonas*, cold shock proteins, *Nicotiana benthamiana*, bacterial effector translocation, plant immunity

## Abstract

**Introduction:**

Plants detect the invasion of microbial pathogens through pathogen-associated molecular patterns (PAMPs). Cold shock proteins (CSPs) are a class of PAMPs specifically recognized by *Solanales* plants. While peptide inoculation studies have revealed the effects of CSPs, their *in vivo* roles remain poorly understood.

**Methods:**

A model system involving the interactions between *Pseudomonas fluorescens* and *P. syringae* pv. *tomato* DC3000 with *Nicotiana benthamiana* has been widely used to investigate the molecular mechanism of plant-microbe interactions. Here, we employed this model system to explore the *in vivo* roles of CSPs in modulating plant immunity by multiple genetic approaches.

**Results:**

Our findings revealed that three highly-conserved CSPs were identified in *Pseudomonas* strains. Transient expression of these CSPs neither induced reactive oxygen species (ROS) production nor suppressed the hypersensitive response (HR) in *N. benthamiana*, however, it restricted bacterial effector translocation. Genetic analysis revealed that these CSPs did not contribute to the ROS burst or HR inhibition *in vivo* but were functionally redundant in suppressing effector translocation in a flagellin (FliC)-independent manner. Furthermore, we demonstrated that the suppression of effector translocation mediated by CSPs was less pronounced compared to that triggered by FliC. Additionally, inoculation with csp15 and csp22 epitopes triggered the pattern-triggered immunity-associated suppression of effector translocations.

**Discussion:**

This study revealed the redundant roles of CSPs in suppressing bacterial effector translocation *in vivo*, providing deep insights into the PTI elicited by cytoplasmic bacterial proteins.

## Introduction

Plant defenses against microbial pathogens are activated upon detection of infectious agents. A series of pathogen-associated molecular patterns (PAMPs) are recognized by pattern recognition receptors (PRRs) to activate pattern-triggered immunity (PTI) (Wang L. et al., 2016). PTI represents the first line of plant innate immunity, offering broad-spectrum resistance against pathogen invasion. Adapted pathogens deploy effector proteins that undermine PTI and promote pathogen proliferation. In response, plants have evolved nucleotide-binding leucine-rich repeat proteins that recognize specific effector proteins, initiating effector-triggered immunity, a robust immune response along with the emergence of a hypersensitive response (HR) to restrict pathogen propagation. A key indicator of PTI activation is the accumulation of reactive oxygen species (ROS), typically observed minutes after epitope inoculation in plant leaves and several hours following bacterial infiltration (Boller and Felix, 2009; Nguyen et al., 2010). Functional PTI can also be detected approximately 6 h after infiltrating *Nicotiana benthamiana* leaves with non-virulent bacteria, as evidenced by the suppression of pathogen effectors translocation, pathogen growth, or HR induction (Wei et al., 2013).

Many bacteria produce small cold-shock proteins (CSPs) to counteract the damaging effects of temperature downshifts and protect cells (Charollais, 2004; Keto-Timonen et al., 2016). CSPs are small nucleic acid-binding proteins, approximately 70 amino acids in length, and are highly-conserved across bacteria. Although CSPs are primarily known for their role in the cold-shock response, recent studies suggest that they are also involved in diverse biological processes. CspD from *Escherichia coli* suppresses DNA replication and its overexpression is lethal to cells (Yamanaka and Inouye, 2001; Uppal et al., 2014). In *Clostridium botulinum* ATCC3502, CspB and CspC are crucial for survival under NaCl, pH, or ethanol stress, and mutations in *cspA* and *cspC* hinder flagella formation and motility (Derman et al., 2015). Additionally, *cspA* in *Brucella melitensis* is critical in regulating virulence and metabolism (Wang Z. et al., 2016). Furthermore, CSPs play crucial roles in plant-microbe interactions. A CSP from *Staphylococcus aureus* has been identified as a PAMP specifically recognized by tomato (*Solanum lycopersicum*), tobacco (*Nicotiana tabacum*), and potato (*Solanum tuberosum*) (Felix and Boller, 2003). Although peptide-inoculation studies have shown that CSPs manipulate plant immunity, the mechanisms underlying CSP–plant interaction *in vivo* remain poorly understood.

A model system involving the interactions between *Pseudomonas fluorescens* and *P. syringae* pv. *tomato* DC3000 (hereafter, *Pst* DC3000) with *N. benthamiana* has been widely used to investigate the molecular mechanism of plant-microbe interactions, providing an ideal system to explore the roles of CSPs *in vivo*. *Pst* DC3000 is a model pathogen for *Arabidopsis*, tomato, and *N*. *benthamiana* (when the avirulent gene *hopQ1*-1 is deleted) and is equipped with a functional Type III Secretion System (T3SS) which translocates virulent effectors into plant cells to cause disease. *P. fluorescens* Pf0-1 is a non-virulent strain that is deficient in a typical T3SS apparatus but can induce PTI in plants. However, Pf0-1 can translocate type III effectors when carrying the functional *hrp*/*hrc* (hypersensitive response and pathogenesis/conserved) T3SS gene cluster on the cosmid pHIR11 (Mastropaolo et al., 2012; Wei et al., 2013; Ngou et al., 2021; Jayaraman et al., 2023).

In this study, we aimed to explore the role of CSPs in modulating plant immunity in the interactions between *Pseudomonas* and *N. benthamiana*. Our findings demonstrate that CSPs play a role in modulating plant immunity through a genetic strategy, providing deep insights into the PTI elicited by cytoplasmic bacterial proteins.

## Materials and methods

### Strains, plasmids, primers, peptides, and plant materials

The strains, plasmids, and primers used in this study are listed in Supplementary Tables S1–S3, respectively. All peptides used were synthesized by GenScript and are summarized in Supplementary Table S4. *P. syringae* and *P. fluorescens* were cultured in King’s medium B (KB) (King et al., 1954), *Agrobacterium tumefaciens* was grown in Luria-Bertani (LB) broth at 28°C, and *E. coli* was grown in LB broth at 37°C. The following antibiotic concentrations were used: ampicillin, 50 mg/mL; rifampicin, 50 mg/mL; spectinomycin 50 mg/mL; and tetracycline 20 mg/mL. *N. benthamiana* plants were grown in a chamber maintained at 16 h light/8 h dark, 65% humidity, and temperatures of 24°C during the day and 22°C at night.

### ROS assay

ROS measurements were performed as previously described (Wei et al., 2013), with minor modifications. Briefly, fresh Pf0-1 and its derivatives were infiltrated into *N. benthamiana* leaves. The concentrations of bacterial suspensions are indicated in the figure legends. Leaf disks from the infiltrated area were excised 15 h post-inoculation (hpi) (36 hpi for transient expression) and placed into the wells of a 96-well plate pre-supplied with 100 μL of sterile water. Then, 100 μL of 0.5 mM L-012 (Wako, Japan) in 10 mM morpholinepropanesulfonic acid–KOH buffer (pH 7.4) was added to each well. ROS accumulation was measured by monitoring chemiluminescence using a microplate reader (Tecan, Switzerland).

### *Agrobacterium*-mediated transient expression

Transient expression was performed as previously described (Wei et al., 2013). Briefly, transformed *Agrobacterium* strains were resuspended in 10 mM MES (pH 5.5) containing 200 μM acetosyringone to the desired optical density and incubated at 28°C for 3 h. The suspension was then infiltrated into *N. benthamiana* leaves using a 1 mL sterile syringe (without the needle). The inoculated plants were maintained in a greenhouse for subsequent assays.

### Challenged-inoculation HR and bacterial growth assays

The challenged-inoculation assay was performed as previously described (Wei et al., 2015). Briefly, fresh streaks of Pf0-1 and its derivatives, as well as *Pst* DC3000, were prepared from isolated colonies and grown overnight at 28°C on KB plates. For the HR suppression assay, Pf0-1 and its derivatives were pre-inoculated into *N. benthamiana* leaves at 2 × 10^8^ CFU/mL. Six hours later, *Pst* DC3000 was infiltrated into the pre-treated areas at 2 × 10^7^ CFU/mL. Images were captured 2 d after *Pst* DC3000 infiltration. For the bacterial growth assay, Pf0-1 and its derivatives were inoculated into *N. benthamiana* leaves at 4 × 10^7^ CFU/mL. Six hours later, the strain *Pst* DC3000Δ*hopQ1–1* was infiltrated into the pre-treated leaves at 5 × 10^5^ CFU/mL. Leaf disks were harvested 4 d post-inoculation (dpi) to assess the bacterial population.

### Effector translocation assay

The effector translocation assay was performed as previously described (Wei et al., 2013), with minor modifications. Briefly, bacterial infiltrations were performed using varying inoculum levels, as indicated in the figure legends. Seventy-two hours later, Pf0-1 (pCPP6225 + pCPP3221) was inoculated into *N. benthamiana* leaves transiently expressing CSPs or FliC derivatives, and leaf disks were collected 6 h after infiltration. For the challenge effector translocation assay, Pf0-1 (pCPP6225 + pCPP3221) or Pf0-1 (pCPP5316) was challenge-inoculated 6 h after pre-inoculation with Pf0-1 and its derivatives, and the leaf disks were harvested 6 hpi. To assess suppression of effector translocation induced by flg22, csp22, and csp15, peptides were infiltrated into *N. benthamiana* leaves at a concentration of 1 μM each. At 6 hpi, *Pst* DC3000 (pCPP3221) expressing AvrPto-Cya at 1 × 10^7^ CFU/mL was challenge-inoculated into the plant leaves. Leaf disks were harvested 6 hpi, excised using a 1.0-cm-diameter cork borer, flash-frozen in liquid nitrogen, and ground in 250 μL of 0.1 M HCl. cAMP levels were measured using a Correlate-EIA cAMP immunoassay kit (Enzo, United States), according to the manufacturer’s instructions.

### RNA preparation and qRT-PCR

The peptides were infiltrated into *N. benthamiana* leaves at a concentration of 100 nM. Three leaf disks were collected from the infiltrated area 6 hpi and ground in liquid nitrogen. Total RNA was isolated using the Plant RNA Kit (R6827-02, OMEGA, United States), following the manufacturer’s instructions. cDNA was synthesized using the FastKing RT kit (KR116-02; TIANGEN, China). Real-time PCR reactions were performed using the SYBR^®^ Green Premix Pro Taq HS qPCR Kit (AG11701, Accurate Biology, China) and ROX Reference Dye (4 mM) (AG11710, Accurate Biology, China). The expression levels of *WRKY22* and *ERF1a* were quantified using the qPCR primers listed in Supplementary Table S1. *NbEF1α* was used as a reference gene.

### Accession numbers

The protein sequences used in this study are available under the following accession numbers: AAO55887.1 (CspA for *Pst* DC3000), ABA73672.1 (*P. fluorescens*), AAZ34509.1 (*P. savastanoi* pv. *phaseolicola* 1448A), UZD98366.1 (*P. corrugata* B21-055), UUI32708.1 (*P. putida* ATCC 12633), AAO57601.1 (CspB for *Pst* DC3000), ABA72944.1 (*P. fluorescens*), AAZ36544.1 (*P. savastanoi* pv. *phaseolicola* 1448A), UZD96690.1 (*P. corrugata* B21-055), UUI33322.1 (*P. putida* ATCC 12633), AAO54799.1 (CspC for *Pst* DC3000), ABA76132.1 (*P. fluorescens*), AAZ35758.1 (*P. savastanoi* pv. *phaseolicola* 1448A), UZD94220.1 (*P. corrugata* B21-055), and UUI36241.1 (*P. putida* ATCC 12633).

### Statistical analyses

Statistical analyses were performed using IBM SPSS Statistics software. Different letters indicate significant differences (*p* < 0.05 by one-way ANOVA).

## Results

### Three CSPs from *Pst* DC3000 are highly conserved across strains of *Pseudomonas*

**Figure 1:**
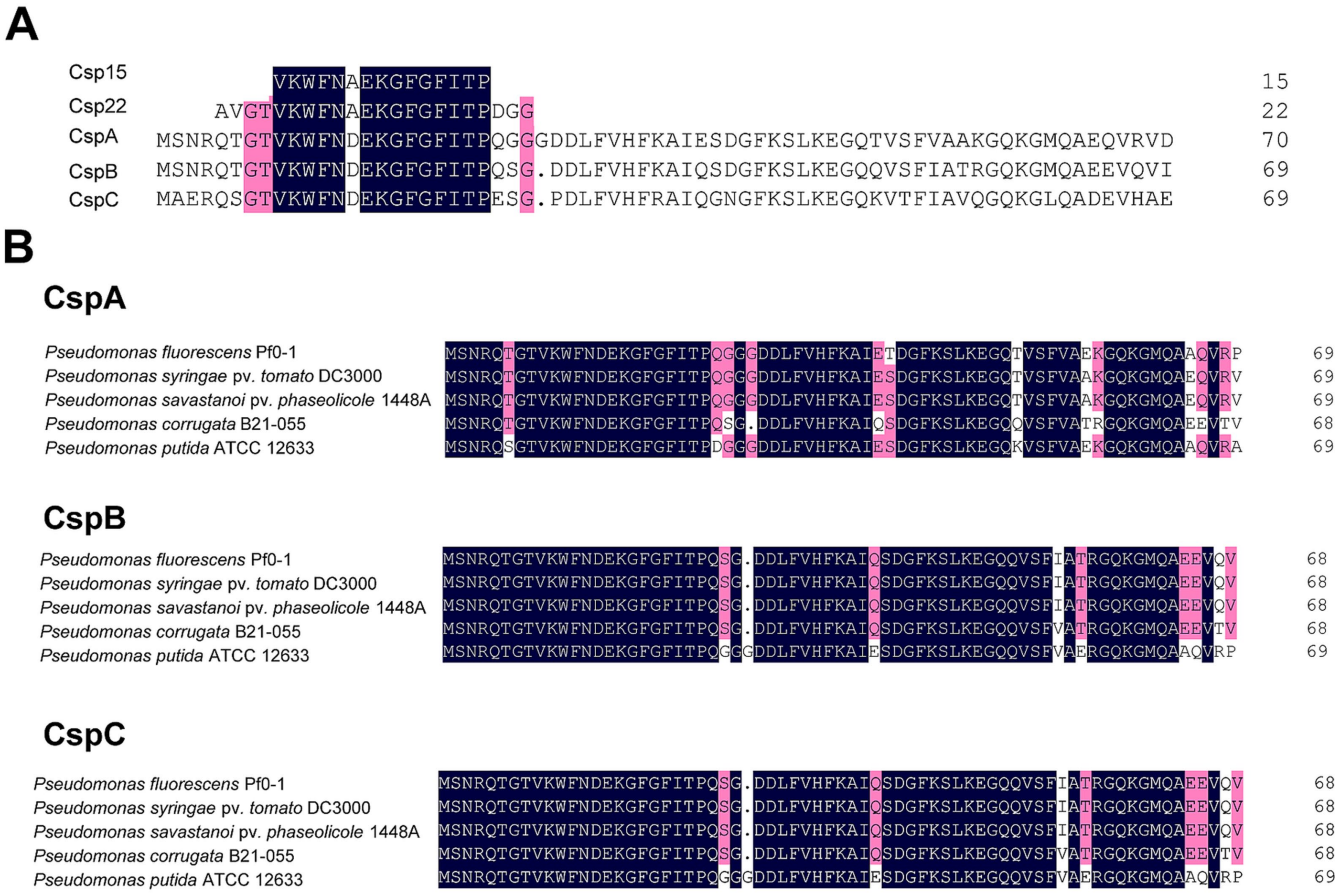
Sequence alignment of CSPs from *Pseudomonas* strains. **(A)** Alignment of CspA, CspB, and CspC of *Pst* DC3000 with csp15 and csp22 peptides. **(B)** Alignment of CspA, CspB, and CspC in plant-and environment-associated *Pseudomonas* strains. Amino acids of CSPs were downloaded from NCBI, while the sequences of csp15 and csp22 were obtained from the previous report (Wang L. et al., 2016). Sequence alignment was performed by DNAMAN. The point indicates the absence of amino acids at that position. Dark blue represents identical amino acids, while red indicates similar amino acids sharing homology between 75 and 100%, respectively.

To identify the CSPs in *Pst* DC3000, we performed a BLASTp analysis using the amino acids of csp15 and csp22—two peptides with PAMP activity that contain the RNP-1 epitope of CSP (Felix and Boller, 2003; Wang L. et al., 2016). Three proteins, CspA (AAO55887.1), CspB (AAO57601.1), and CspC (AAO54799.1), were identified by means of this method. Sequence alignment revealed that all three proteins were highly conserved in *Pst* DC3000, differing by only one amino acid from csp15 and five amino acids from csp22 (Figure 1A). We also performed multiple alignments of the three CSPs with the CSPs of other plant and environment associated-*Pseudomonas* strains, including the well-studied strain *P. fluorescens* Pf0-1. The results showed that these three CSPs were well conserved; CspA shares 94% homology with its homologs, CspB shares 95.43%, and CspC shares 96.86%. Collectively, these findings suggest that these CSPs may exhibit similar functions across diverse *Pseudomonas* strains (Figure 1B).

### *Agrobacterium*-mediated transient expression of CSPs from *Pst* DC3000 suppresses effector translocation

**Figure 2:**
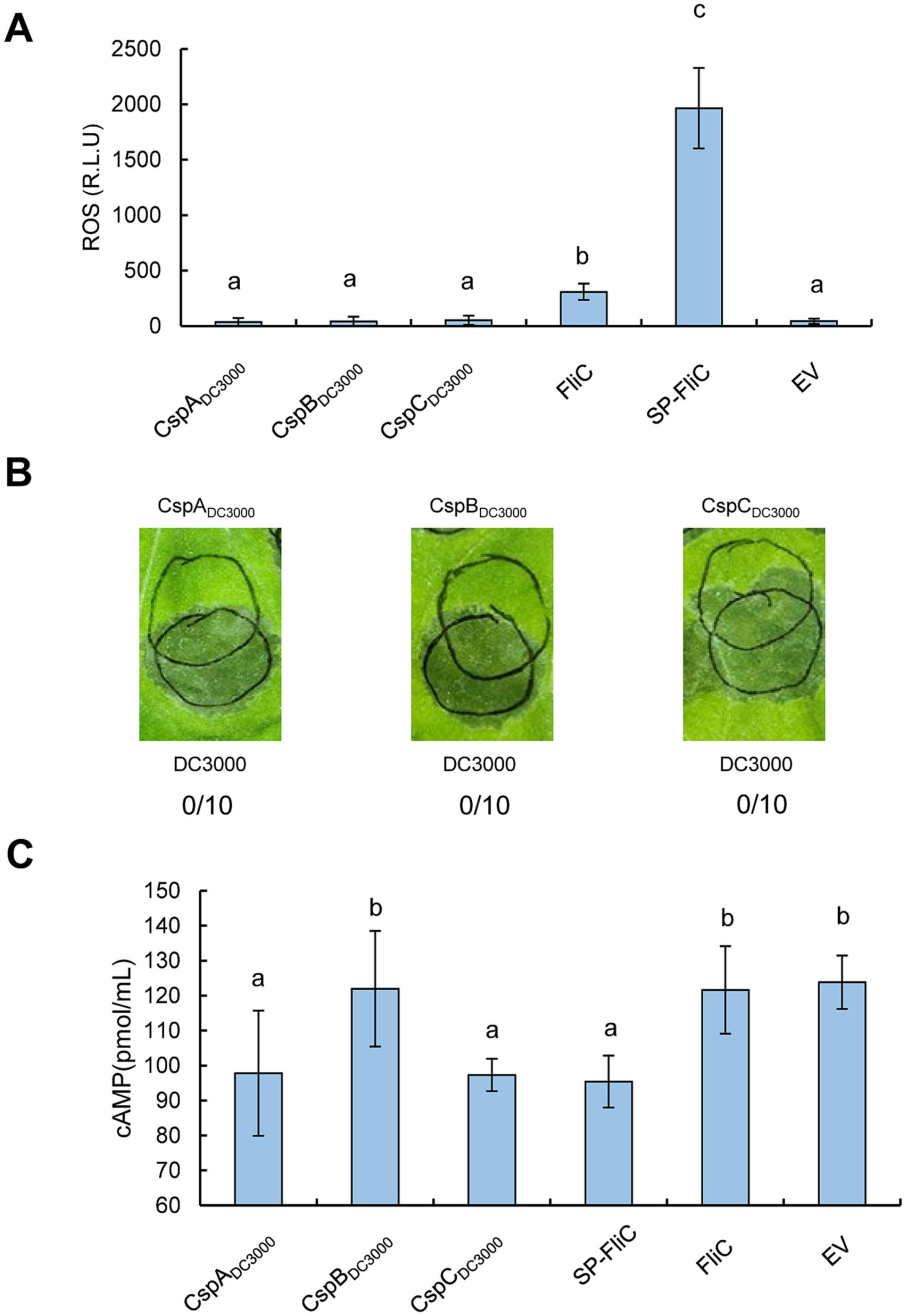
Assessments of PTI induction activities of CSPs from *Pst* DC3000 by *Agrobacterium*-mediated transient expression in *N. benthamiana* leaves. **(A)** CSPs cannot induce ROS production when transiently expressed in *N. benthamiana* leaves. CSPs from *Pst* DC3000 were *Agrobacterium*-mediated transiently expressed in *N. benthamiana* leaves at 2 × 10^8^ CFU/mL, leaf disks were collected at 36 hpi to monitor ROS accumulation. **(B)** CSPs cannot suppress HR when transiently expressed in *N*. *benthamiana* leaves. The three CSPs were *Agrobacterium*-mediated transiently expressed at 2 × 10^8^ CFU/mL prior to an overlapping infiltration of wild-type *Pst* DC3000 at 4 × 10^7^ CFU/mL. The upper circles indicate transient expression inoculation, while the lower circles indicate infiltration with *Pst* DC3000. Leaves were photographed 48 h after the challenge inoculation. The number of times each test CSP induced PTI as a fraction of number of times tested are shown below each photograph, respectively. **(C)** CSPs suppress effector translocation when transiently expressed in *N*. *benthamiana* leaves. *Agrobacterium* cells harboring expression vectors were infiltrated with *N. benthamiana* leaves at 2 × 10^8^ CFU/mL, and challenged 72 h later with an inoculation of Pf0-1 (pCPP6225 + pCPP3221) at 4 × 10^7^ CFU/mL, leaf disks were harvested at 12 hpi to determine the cAMP level. EV indicates empty vector. Different letters indicate significant differences (*p* < 0.05 by one-way ANOVA), respectively. All experiments were repeated three times with similar results.

To determine whether the CSPs of *Pst* DC3000 modulate plant immunity *in vivo*, we cloned the CDSs of CspA, CspB, and CspC and transiently expressed them in *N. benthamiana* leaves under the control of the 35S promoter. First, we assessed whether the CSPs stimulated ROS accumulation. It has been well established that FliC was a major PAMP of *Pseudomonas* recognized by *N. benthamiana*, we therefore took advantage of FliC and FliC fused with the PR1a signal peptide (SP-FliC) as controls. As previously reported, FliC and SP-FliC induced weak and strong ROS bursts, respectively (Wei et al., 2013). However, CspA, CspB, and CspC did not stimulate ROS accumulation (Figure 2A). In the HR suppression analysis, *Agrobacterium tumefaciens* harboring pEarleyGateS101 carrying *csp* genes were inoculated into *N. benthamiana* leaves at 2 × 10^8^ CFU/mL and then incubated for 48 h before partially overlapping challenge inoculation with a suspension of 4 × 10^7^ CFU/mL of wild-type *Pst* DC3000. None of the CSPs inhibited the HR induced by *Pst* DC3000 (Figure 2B). The activation of PTI inhibits the translocation of effector proteins (Anderson et al., 2014). Therefore, we next investigated whether CSPs suppressed effector translocation by employing Pf0-1 harboring plasmids pCPP3221 expressing AvrPto-Cya and pCPP6225 (a derivative of T3SS^+^ pHIR11 with an unmarked deletion of the genes encoding the effector HopA1 and its chaperone ShcA). *N. benthamiana* leaves were infiltrated with *A. tumefaciens* cells and then challenged 72 h later with an overlapping inoculation of Pf0-1 (pCPP6225 + pCPP3221) for the Cya-based translocation assay, in which the translocation of effectors into plant cells can be clearly detected on the basis of the Cya activity as a high production of cAMP (Mukaihara and Tamura, 2009). As shown in Figure 2C, the expression of CspA and CspC, but not CspB, significantly suppressed AvrPto-Cya translocation, as well as SP-FliC, compared to EV and FliC, indicating that CspA and CspC of *Pst* DC3000 suppress effector translocation when transiently expressed in *N. benthamiana* leaves. Collectively, these results suggest that the CSPs of *Pst* DC3000 can suppress effector translocation in *N*. *benthamiana* leaves.

### CSPs from *Pseudomonas fluorescens* Pf0-1 are redundant in suppressing effector translocation independent of FliC

**Figure 3:**
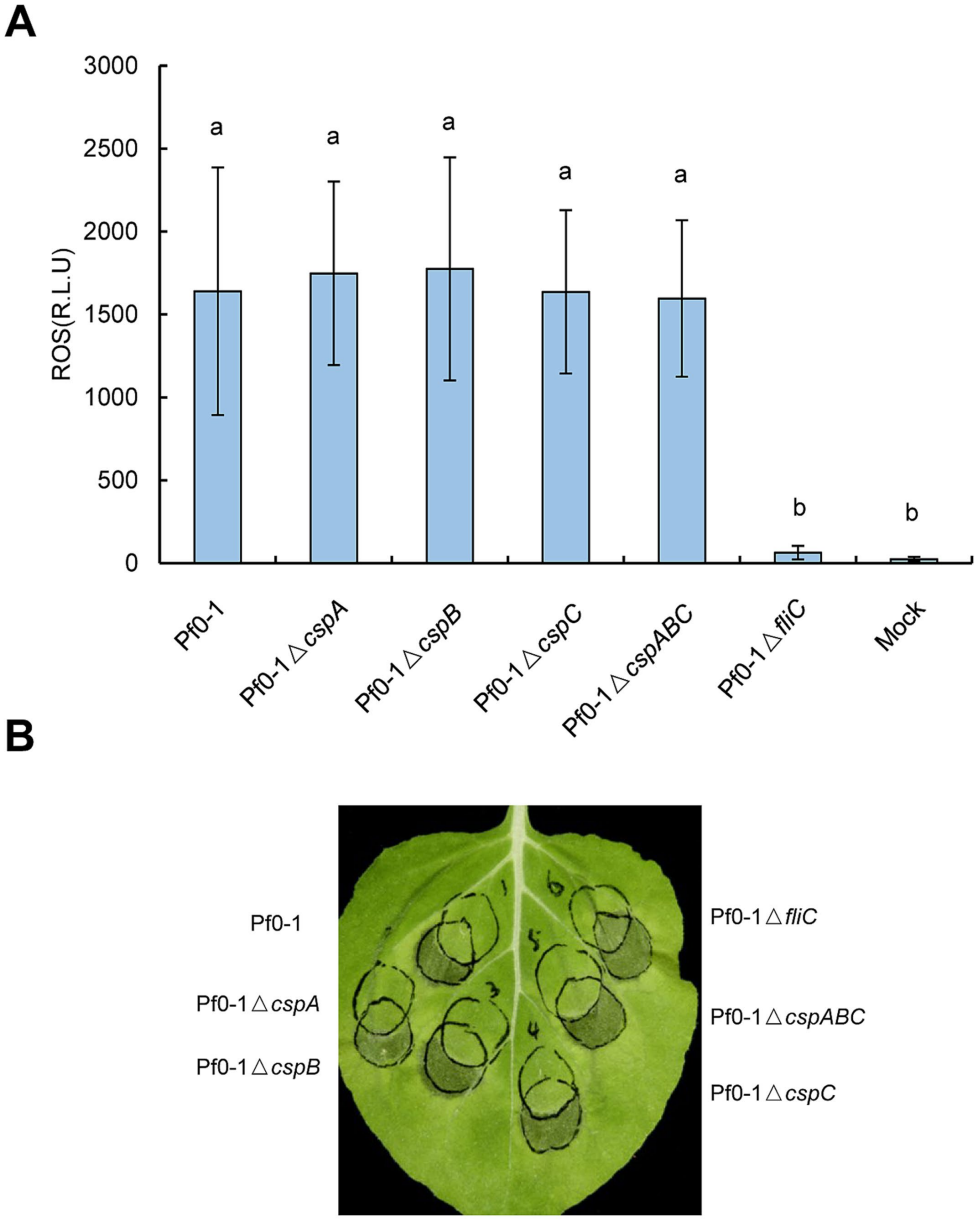
*Pseudomonas* CSPs have no contributions to the ROS burst or HR inhibition *in vivo*. **(A)** Mutations of *csp* genes have no impact on ROS accumulation. Pf0-1 and its derivatives were inoculated into *N. benthamiana* leaves at 2 × 10^8^ CFU/mL, leaf disks were collected at 15 hpi to monitor the ROS level. Different letters indicate significant differences (*p* < 0.05 by one-way ANOVA), respectively. **(B)** Mutations of *csp* genes of Pf0-1 did not impair HR suppression ability. Pf0-1 and its derivatives were inoculated into *N. benthamiana* leaves at 2 × 10^8^ CFU/mL, then 6 h later, *Pst* DC3000 was overlapped infiltrated with the pre-treated areas at 2 × 10^7^ CFU/mL. For the three treatments on the left side of the leaf, the right or upper circles represent inoculation with Pf0-1 or its derivatives; for the three treatments on the right side of the leaf, the left or upper circles indicate inoculation with Pf0-1 derivatives. The pictures were photographed 48 h after challenge infiltration. All experiments were repeated three times with similar results.

To further probe into the function of CSPs *in vivo* and to circumvent the effects of effectors, we employed *P. fluorescens* Pf0-1 in subsequent assays. Mutants lacking individual CSPs (Δ*cspA*, Δ*cspB*, or Δ*cspC*) and the triple mutant Δ*cspABC* were generated and subjected to ROS burst assay. The Δ*fliC* mutant served as a positive control, while 10 mM MgCl_2_ served as a reagent control. As expected, Pf0-1 strongly induced ROS accumulation compared to the mock treatment, whereas Δ*fliC* induced negligible ROS production (Figure 3A). ROS levels in *N*. *benthamiana* leaves treated with Δ*cspA*, Δ*cspB*, Δ*cspC*, or the triple mutant Δ*cspABC* were comparable to those in leaves treated with Pf0-1, suggesting that deleting the three CSPs could not impair the ROS burst (Figure 3A).

**Figure 4:**
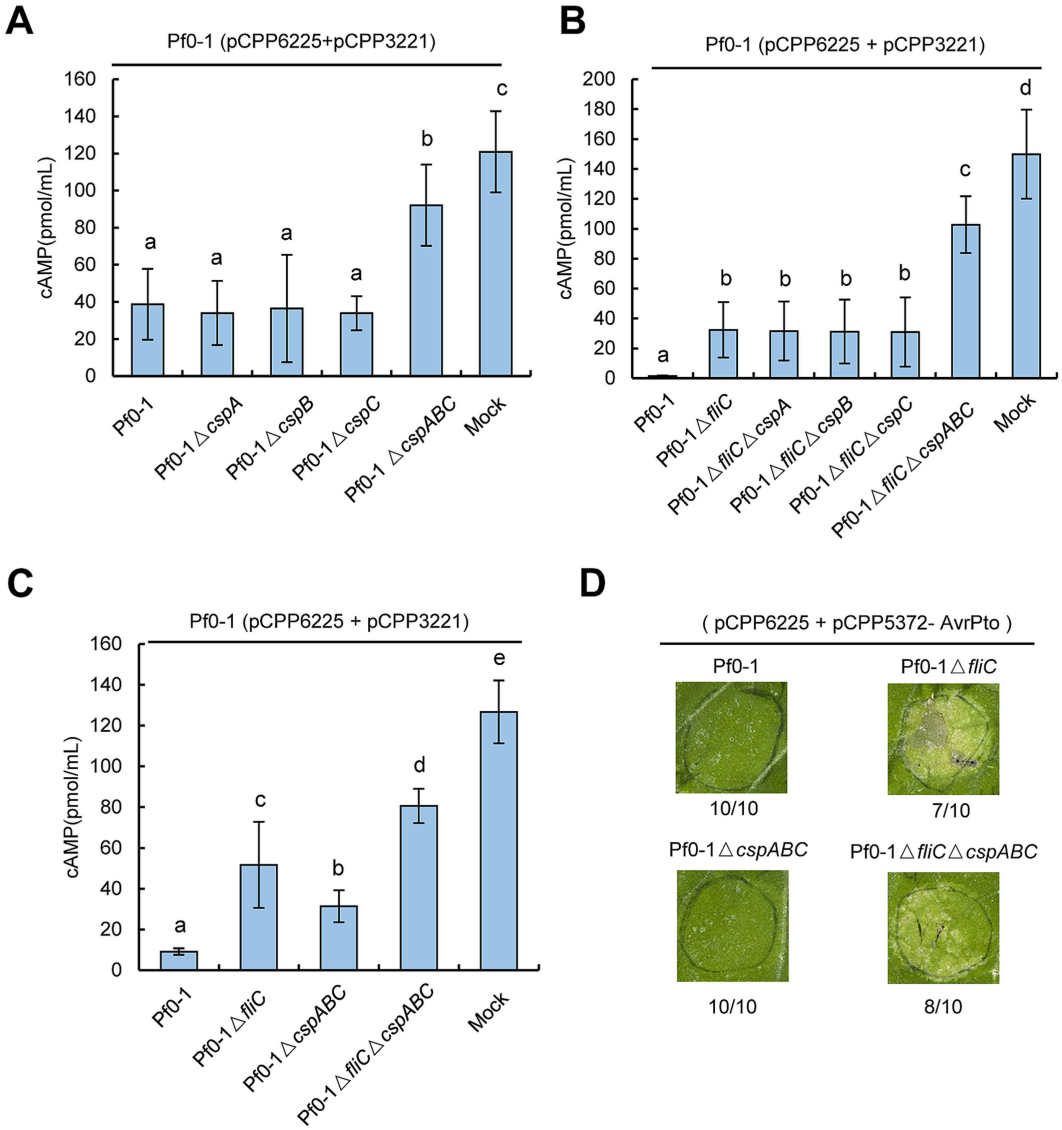
*Pseudomonas* CSPs share functional redundancy in restricting effector translocation in a FliC-independent manner. **(A)** *Pseudomonas* CSPs are redundant in suppressing effector translocation. *P. fluorescens* strains were infiltrated with *N. benthamiana* leaves at 4× 10^7^ CFU/mL and challenged 6 h later with an overlapping inoculation of *P. fluorescens* (pCPP6225 + pCPP3221) expressing AvrPto-Cya at 4 × 10^7^ CFU/mL. At 6 h after the challenge inoculation, leaf disks were collected from areas of overlap between pretreatment and challenge inoculations to determine the cAMP level. **(B)** *Pseudomonas* CSPs suppress effector translocation independent of FliC. *P. fluorescens* strains were infiltrated with *N. benthamiana* leaves at 2 × 10^8^ CFU/mL, challenge inoculation was done as above. **(C)** FliC suppresses more AvrPto translocation than CSPs. *P. fluorescens* strains were infiltrated with *N. benthamiana* leaves at 2 × 10^8^ CFU/mL, challenge inoculation was done as above. **(D)** Mutations of *fliC* but not *csp* genes generate AvrPto-induced chlorosis. Pf0-1 derivatives carrying pCPP6225 and pCPP5372-AvrPto were infiltrated with *N*. *benthamiana* leaves at 5 × 10^8^ CFU/mL. Pictures were photographed at 72 hpi. Relative numbers of chlorosis in inoculated zones are shown. Different letters indicate significant differences (*p* < 0.05 by one-way ANOVA), respectively. All experiments were repeated three times with similar results.

Next, we examined whether the ability of Pf0-1 to suppress HR was associated with CSPs. *N. benthamiana* plants were inoculated with Pf0-1 and its derivatives at 4 × 10^7^ CFU/mL, incubated for 6 h, and then challenge inoculated with wild-type *Pst* DC3000 at 2 × 10^7^ CFU/mL. All treatments, including Pf0-1, Δ*cspA*, Δ*cspB*, Δ*cspC*, or the triple mutant Δ*cspABC*, suppressed the HR induced by *Pst* DC3000, whereas the control Δ*fliC* mutant lost this ability, indicating that mutating these three CSPs does not affect the HR suppression ability of Pf0-1 (Figure 3B). Next, we used Pf0-1 (pCPP6225 + pCPP3221) to investigate whether CSPs play a role in suppressing effector translocation. Pf0-1 (pCPP6225 + pCPP3221) was infiltrated into *N. benthamiana* leaves 6 h after inoculation with Pf0-1 and its derivatives to measure cAMP levels. The results showed that cAMP levels induced by Pf0-1, Δ*cspA*, Δ*cspB*, or Δ*cspC* treatments were analogous, but were significantly lower than the mock treatment. However, Δ*cspABC* inoculation generated notably higher cAMP levels than Pf0-1, Δ*cspA*, Δ*cspB*, or Δ*cspC* treatments, suggesting that CspA, CspB, and CspC of Pf0-1 were functionally redundant in mitigating the effector injection restriction (Figure 4A).

FliC is a major *Pseudomonas* PAMP recognized by *N. benthamiana.* Therefore, we investigated whether the ability of CSPs to suppress effector translocation was related to FliC. We constructed Pf0-1Δ*fliC* mutants with individual or combined deletions of *cspA*, *cspB*, and *cspC*. Effector translocation assays showed that Δ*fliC* induced higher cAMP levels than Pf0-1 (Figure 4B). Moreover, cAMP levels induced by Pf0-1Δ*fliC*, Δ*fliC*Δ*cspA*, Δ*fliC*Δ*cspB*, or Δ*fliC*Δ*cspC* were similar, but significantly lower than those induced by Δ*fliC*Δ*cspABC* (Figure 4B). These data indicate that CSPs are redundant in suppressing effector translocation independent of FliC.

### CSPs-mediated suppression of effector translocation is modest compared to that triggered by FliC

To compare the relative contributions of FliC and CSPs to effector translocation inhibition, we conducted further assays. *N. benthamiana* leaves were inoculated with Pf0-1 and its derivatives at 2 × 10^8^ CFU/mL and challenged 6 h later with Pf0-1 (pCPP6225 + pCPP3221) at 4 × 10^7^ CFU/mL. Effector translocation assays showed that Pf0-1Δ*fliC* induced significantly higher cAMP levels than Pf0-1Δ*cspABC*, indicating that FliC inhibits effector translocation more effectively than CSPs (Figure 4C). Furthermore, we transformed pCPP5372-AvrPto into Pf0-1 derivatives carrying pCPP6225, infiltrated them into *N. benthamiana* leaves at 5 × 10^8^ CFU/mL, and kept the plants in the chamber for 3 days. The results showed that Δ*fliC* and Δ*fliC*Δ*cspABC*, but not Δ*cspABC*, caused AvrPto-induced chlorosis, suggesting that mutation of FliC allowed more AvrPto translocation than CSPs (Figure 4D). Additionally, Δ*fliC*Δ*cspABC* induced significantly higher cAMP levels than Δ*fliC* and Δ*cspABC*, further supporting the conclusion that CSPs suppress effector translocation independently of FliC (Figure 4C). We further analyzed the effect of these mutations on HopA1 translocation. Pf0-1 was transformed with pCPP5316 carrying the functional T3SS cluster and HopA1-Cya, and challenge-inoculation effector translocation assays were performed. The results showed that Δ*fliC* induced significantly higher cAMP levels than Pf0-1, but lower than Δ*fliC*Δ*cspABC*, suggesting that both *fliC* and *cspABC* mutations increase HopA1 translocation (Supplementary Figure S1). We also investigated whether mutations in CSPs alleviated PTI-associated inhibition of challenge-inoculated pathogen growth. Pf0-1 and its derivatives were infiltrated into *N. benthamiana* leaves at 4 × 10^7^ CFU/mL and incubated for 6 h before challenge inoculation with *Pst* DC3000Δ*hopQ1–1* at 5 × 10^5^ CFU/mL. The results showed that at 4 dpi, no significant difference in pathogen population suppression was observed between the CSP-deleted mutants and Pf0-1 or Pf0-1Δ*fliC* (Supplementary Figure S2). Collectively, these results suggest that the suppression of effector translocation mediated by CSPs is moderate compared to that mediated by FliC.

### csp22 and csp15 suppress effector translocation in *Nicotiana benthamiana*

**Figure 5:**
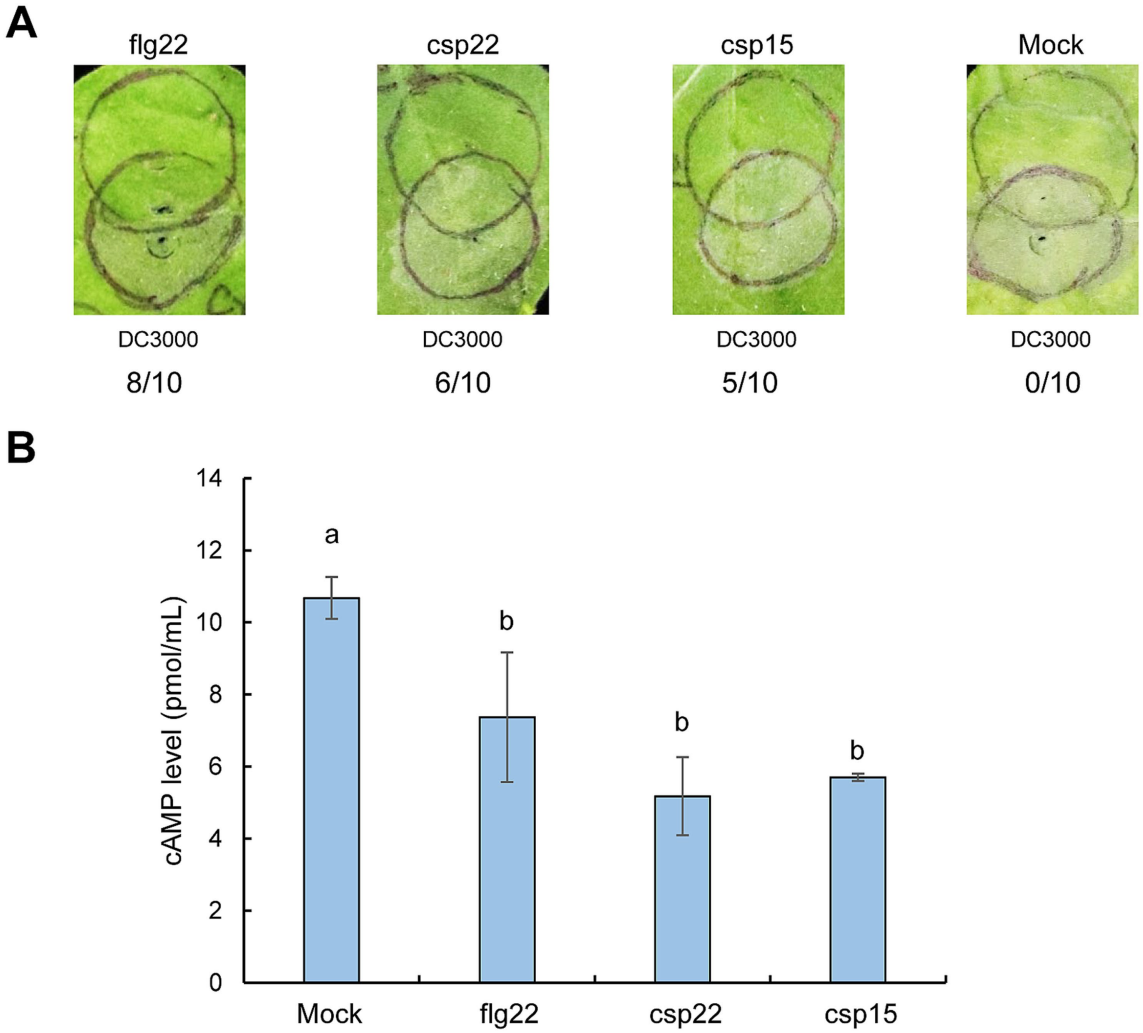
Inoculation of csp22 and csp15 restricts effector translocation in *N. benthamiana* plants. **(A)** Inoculation of csp22 and csp15 suppresses HR induced by *Pst* DC3000. Peptides were infiltrated with *N. benthamiana* leaves at a concentration of 1 μM. At 6 hpi, *Pst* DC3000 suspension was challenge-inoculated into plant leaves at 5 × 10^6^ CFU/mL. Pictures were photographed at 72 h post final inoculation. The upper circles were inoculated with peptides, while the lower circles were infiltrated with *Pst* DC3000. The number of times each test peptide induced PTI as a fraction of number of times tested are shown below each photograph, respectively. **(B)** Inoculation of csp22 and csp15 suppresses effector translocation. The inoculation of peptides was performed as above and challenge 6 h later with *Pst* DC3000 (pCPP3221) expressing AvrPto-Cya at 1 × 10^7^ CFU/mL. At 6 h after the challenge inoculation, leaf disks were collected from areas of overlap between pretreatment and challenge inoculations to determine the cAMP level. Different letters indicate significant differences (*p* < 0.05 by one-way ANOVA), respectively. All experiments were repeated three times with similar results.

To further validate the role of CSPs in restricting effector translocation, we synthesized csp22 and csp15 peptides, which represent the RNP-1 epitope of CSP with PAMP activity as well as the conserved flg22 epitope. We aimed to test whether inoculation with these peptides could induce PTI-associated suppression of effector translocation by assessing their ability to inhibit HR and the cAMP levels produced by Cya fusion. We found that HR was nearly eliminated in *N. benthamiana* leaves pre-infiltrated with flg22 following challenge inoculation with *Pst* DC3000, whereas it was partially eliminated in leaves pre-infiltrated with csp22 or csp15 (Figure 5A). Moreover, pre-infiltration with flg22, csp22, or csp15 resulted in significantly lower cAMP levels than the mock treatment, indicating that these peptides effectively suppressed effector translocation in *N*. *benthamiana* (Figure 5B). Additionally, inoculation with these peptides upregulated the PTI marker gene—*WRKY22*—and the ethylene-related marker gene—*ERF1a* —consistent with previous reports (Felix and Boller, 2003; Saur et al., 2016) (Supplementary Figure S3). Collectively, these findings suggest that csp22 and csp15 trigger PTI-associated suppression of effector translocation.

## Discussion

In this study, we employed genetic approaches to investigate the role of *Pseudomonas* CSPs in modulating plant immunity. Multiple alignments revealed that the CSPs were highly conserved in pathogenic and nonvirulent *Pseudomonas* strains, consistent with a recent finding suggesting that these CSPs shared similar functions (Chen et al., 2024) (Figure 1B). Further genetic assessments indicated that CSPs from *Pst* DC3000 and Pf0-1 did not contribute to ROS burst or the suppression of HR induced by *Pst* DC3000 *in vivo*. However, CSPs were redundant in suppressing bacterial effector translocation in a FliC-independent manner (Figures 2–4). Additionally, inoculation with csp15 and csp22 peptides suppressed effector translocation (Figure 5B). These findings suggest that CSPs from different bacterial species are functionally conserved in suppressing bacterial effector translocation.

Over two decades ago, a highly conserved CSP was identified as a PAMP that is recognized by the immune system of *Solanales* plants (Felix and Boller, 2003). Subsequent studies identified two PRRs—CORE in tomato and NbCSPR in *N. benthamiana*—that recognize the csp22 epitope (Saur et al., 2016; Wang et al., 2016). Stable transgenic *A. thaliana* plants expressing *NbCORE* or *NbCSPR* exhibited stronger resistance to *Pst* DC3000, implying that *Pseudomonas* CSPs can be recognized by these receptors in *N. benthamiana* plants *in vivo* (Saur et al., 2016; Wang et al., 2016). However, genetic analysis in the current study showed that *Pseudomonas* CSPs did not stimulate ROS production or suppress HR, likely due to the age-dependent low expression of *NbCORE* and *NbCSPR* in 4-week-old *N. benthamiana* plants (Figures 2A,B, 3) (Saur et al., 2016; Wang et al., 2016). Transient expression of CspA and CspC from *Pst* DC3000 restricted effector injection; a result similar to that observed when FliC was transiently expressed in *N. benthamiana* leaves (Figure 2C). This finding suggests that some PAMPs, when strongly expressed under the control of the 35S promoter, may leak from the cytoplasm, enabling extracellular perception by PRRs (Wei et al., 2013). We hypothesized that the transient expression of *cspB* might be too weak to allow the leakage of CspB from the cytosol, accounting for the absence of restriction on effector translocation in our study (Figure 2C). Although *Agrobacterium*-mediated transient expression has been extensively utilized in exploring the biological function of protein *in planta*, there are still certain limitations in yield and sustainability of expressed proteins. Furthermore, the efficiency of protein expression is affected by the concentration and timing of the inoculation. Therefore, further investigation into the CSPs-triggered PTI responses in the stable expression system of plants will provide deeper insights into the roles of CSPs in plant-microbe interactions.

Gene family expansion is widespread across bacterial genomes and often results in functionally conserved members (Hahn et al., 2007; Collins et al., 2011). The flg22 epitopes from commensal bacteria of *Arabidopsis thaliana* induced similar immune responses (Colaianni et al., 2021). Similar phenomenon was observed in our study which demonstrates that conserved *Pseudomonas* CSPs share functional redundancy in suppressing effector translocation, which is a critical step for bacterial pathogenesis (Figure 4A). Furthermore, we established that CSPs-and FliC-mediated inhibition of effector translocation occured via independent signaling pathways, with FliC suppressing more effector injection than CSPs (Figures 4B,C). This difference in inhibition is likely due to the different localization of FliC and CSPs in bacterial cells; FliC is an extracellular protein more readily exposed to plant membranes to induce immune responses, whereas CSPs are cytoplasmic bacterial proteins, with only a small number present in the secretomes of bacteria (Song et al., 2009; Kühn et al., 2018). This difference in inhibition accounts for FliC being the primary PAMP and the lack of impact from *csp* gene deletion in Pf0-1 on ROS accumulation or HR suppression (Figure 3). Additionally, deletion of *csp* from Pf0-1 or Pf0-1Δ*fliC* did not affect the growth of *Pst* DC3000Δ*hopQ1-1* in the challenge inoculation assay (Supplementary Figure S2). A similar finding, where deletion of *fliC* did not significantly impact the survival of pathogen *Pst* DC3000Δ*hopQ1-1*, was likely due to the high virulence of *Pst* DC3000Δ*hopQ1-1* which masked the minor growth promotion triggered by the defective PTI response (Kvitko et al., 2009). Collectively, our results illustrate significant roles for CSPs in modulating plant immunity, providing deep insights into the mechanisms by which cytoplasmic bacterial proteins-triggered PTI responses.

## Data Availability

The original contributions presented in the study are included in the article/Supplementary material, further inquiries can be directed to the corresponding author.
