# Supplementary material for: Pseudomonas cold shock proteins suppress bacterial effector translocation in Nicotiana benthamiana
Source: Front Microbiol. 2025 Jan 23;16:1539906. doi: 10.3389/fmicb.2025.1539906 (PMC11799257; doi:10.3389/fmicb.2025.1539906)
Supplement: Supplementary file 2 [file Data_Sheet_1.PDF]

Fig. S1

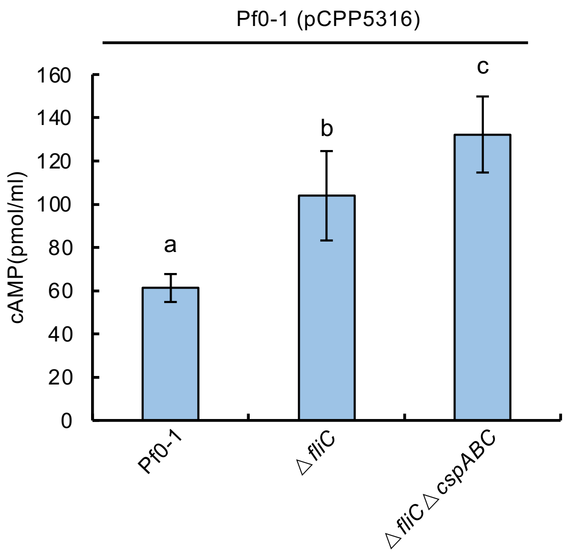

Figure. S1. FliC suppresses more HopA1 translocation than CSPs. Pf0-1 derivatives carrying pCPP5316 esxpressing HopA1-Cya were infiltrated with *N. benthamiana* leaves at $4x10^7$ CFU/mL. At 6 h post-inoculation, two 1.0-cm leaf disks were collected from areas to perform effector translocation assay. Means marked with the same letter are not statistically different at the 5% confidence level based on Duncan's multiple range test. All experiments were repeated three times with similar results.

Fig. S2

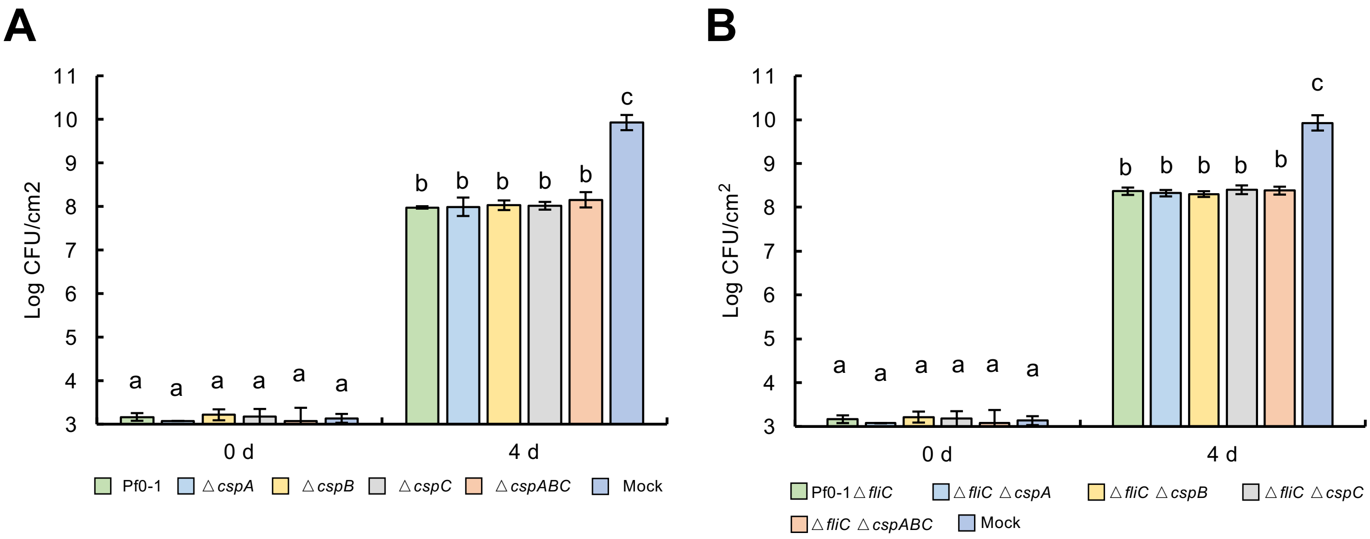

Figure. S2. Mutations of *csp* genes have no impact on PTI-associated inhibition of the growth of challenge-inoculated pathogen. Pf0-1 and its derivatives were infiltrated with *N. benthamiana* plants at $4x10^7$ CFU/mL and incubated 6 hours before challenged inoculation with D3000Δ*hopQ1-1* at $5x10^5$ CFU/mL, leaf disks were harvested 4 dpi to determine the bacterial population. Means marked with the same letter are not statistically different at the 5% confidence level based on Duncan’s multiple range test. All experiments were repeated three times with similar results.

Fig. S3

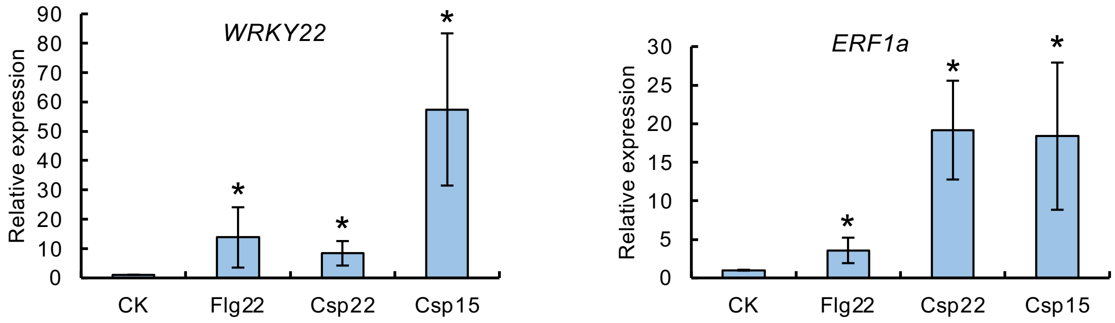

Figure. S3. Inoculation of Flg22, Csp22, and Csp15 altered the expression pattern of *WRKY22* and *ERF1a*. Peptides were infiltrated with *N. benthamiana* leaves at a concentration of 100nM and leaf disks were collected 6 hpi to determine mRNA levels. Data were generated using *NbEF1α* as reference gene. Asterisk represents significant change compared to CK at $P < 0.05$. All experiments were repeated three times with similar results.
